# Publishing a Paper during Residency for Specialists: How Should We Deal with It?

**DOI:** 10.31662/jmaj.2023-0014

**Published:** 2023-05-22

**Authors:** Shigeki Matsubara, Hironori Takahashi

**Affiliations:** 1 Department of Obstetrics and Gynecology, Jichi Medical University, Tochigi, Japan

**Keywords:** certified doctor, obstetrics and gynecology specialist, publishing paper, specialist

## Abstract

Some specialties require publishing a paper as a prerequisite for becoming a Japanese Medical Specialty Board Specialist. Taking obstetrics and gynecology as an example, we wish to describe some concerns about this. Time limitations oblige residents to publish papers in non-PubMed journals with smaller circulations. Once data have been published, later attempts at secondary publication are difficult. This may bury some important data. Requiring an English presentation and writing an English abstract as a prerequisite for a board specialty may be an option to avoid this. Although we believe that the experience of publishing a paper during residency is important, how to deal with this issue needs further consideration.

Writing a paper during residency will promote future research activity, eventually contributing to academia. The Japanese Medical Specialty Board certified 19 clinical specialties. Candidates choose one of these specialties, on entering the senior residency, after which they take a specialist certificate examination. Those who pass the examination become specialists. Although the examination differs among specialties, some require publishing a first author paper as a prerequisite for passing a specialist examination. For example, the Japan Society of Obstetrics and Gynecology (JSOG) requires an obstetrics and gynecology (OBGYN)-resident to publish at least one first author paper as a prerequisite to becoming an OBGYN-Specialist ^(1)^. We have some concerns about this requirement.

First, residents tend to publish papers in journals with less accessibility. We have long been taught that papers should be accessible to a broad readership, and thus submitted to PubMed journals. However, difficulty in publishing papers in PubMed journals during residency forces residents to publish papers in non-PubMed journals, particularly domestic Japanese journals, usually with a small readership. This is because there is no assurance that a manuscript will be accepted by a PubMed journal within a limited period. If not accepted, the resident cannot become an OBGYN-Specialist. During the last 5 years (2018-2022), the Jichi Medical University OBGYN department produced 21 OBGYN-Specialists: only 3 published papers in PubMed-English journals, whereas the remaining 18 published in Japanese non-PubMed journals, of which 15 were published in regional journals. If there had been no time-limitation and residents had been mentored in writing an English paper, studies of many may have been accepted by PubMed journals.

Second, secondary publication of papers already published in domestic Japanese journals is often difficult. This is due to the concern over overlapping publications. After publishing a paper in a domestic journal, its significance might be recognized, making it worthy of secondary publication. The International Committee of Medical Journal Editors has set many regulations regarding “acceptable secondary publications” ^(2)^. Detailed letters are required for the editors of both a domestic (already published) and an international (target submission) journal. The secondary version must reflect interpretations of the primary version: whether any new recognized significance can be described depends on the journal policy. These two considerations may bury data, even though they may contain important findings.

We have two possible solutions. First, an English abstract should also be accepted as a prerequisite. For example, the JSOG annual meeting has an international session. The Japanese OBGYN journal (Acta Obstetrica et Gynaecologica Japonica) provides indexing of JSOG-meeting abstracts ^(3)^. We believe that many other specialties may have Japanese journals with indexing. Alternatively, delivering a presentation at an international meeting should also be a prerequisite: international journals often publish meeting abstracts. These will reach a broader audience. Residents have a chance not only to deliver an English presentation but also write a concise English report. As abstracts are not articles, overlapping publications are avoided. However, some may consider that an abstract is not enough to achieve a specialist certificate.

The second possible solution is to submit to a medical specialty board a paper not yet accepted for publication. It should be evaluated regarding its appropriateness for a specialist certificate. Its later publication should be confirmed. However, these require additional efforts of certification committees.

A statement that publishing a first author paper is required as a prerequisite for the specialist examination is described on the home pages of six specialties. We checked the homepages of the 19 specialties and identified this requirement in the following 6 specialties: pediatrics, neurosurgery, dermatology, otolaryngology, plastic surgery, and OBGYN. Although there have been, to our knowledge, no publications, we believe that the abovementioned problem might also occur in these specialties other than OBGYN. We sent a questionnaire to the directors of these six specialties at Jichi Medical University. Two answered that publishing first author papers was necessary, and another two answered that this was unnecessary. The remaining two had an ambiguous attitude regarding this issue. Irrespective of the pros and cons of publishing papers, all six answered that publishing first author papers as a prerequisite may cause the abovementioned problems.

We would never criticize academic societies, which have their own policies. We propose that designing a study and writing a paper should be considered part of a residency program. However, its actual practice should be widely discussed, irrespective of whether and how this certification system of a specialty involves publishing a paper.

## Article Information

### Conflicts of Interest

None

### Acknowledgement

We thank Reynir Tómas Geirsson (Emeritus Professor, Obstetrics and Gynecology of Women’s Clinic, Landspitali University Hospital, Iceland) and Hitoaki Okazaki (Professor of Medical Education Center, Jichi Medical University, Japan) for their help. We describe our personal impressions and proposals based on the experience of OBGYN professors and not a formal opinion of our Department, Institute, or Prefectural JSOG Committee.

### Author Contributions

SM and HT identified the significance and wrote the manuscript.

### Approval by Institutional Review Board (IRB)

Not needed.

### Informed Consent

Not applicable.

### Patient Anonymity

Not applicable.
